# Neurophysiological Characterization of Subacute Stroke Patients: A Longitudinal Study

**DOI:** 10.3389/fnhum.2016.00574

**Published:** 2016-11-16

**Authors:** Giuseppe Lamola, Chiara Fanciullacci, Giada Sgherri, Federica Bertolucci, Alessandro Panarese, Silvestro Micera, Bruno Rossi, Carmelo Chisari

**Affiliations:** ^1^Unit of Neurorehabilitation, Department of Neuroscience, University of PisaPisa, Italy; ^2^The BioRobotics Institute, Scuola Superiore Sant'AnnaPisa, Italy; ^3^Translational Neural Engineering Lab, Center for NeuroprostheticsLausanne, Switzerland

**Keywords:** stroke, functional recovery, cortical excitability, motor evoked potentials, silent period, intracortical inhibition, unaffected hemisphere, clinical neurophysiology

## Abstract

Various degrees of neural reorganization may occur in affected and unaffected hemispheres in the early phase after stroke and several months later. Recent literature suggests to apply a stratification based on lesion location and to consider patients with cortico-subcortical and subcortical strokes separately: different lesion location may also influence therapeutic response. In this study we used a longitudinal approach to perform TMS assessment (Motor Evoked Potentials, MEP, and Silent Period, SP) and clinical evaluations (Barthel Index, Fugl-Meyer Assessment for upper limb motor function and Wolf Motor Function Test) in 10 cortical-subcortical and 10 subcortical ischemic stroke patients. Evaluations were performed in a window between 10 and 45 days (t0) and at 3 months after the acute event (t1). Our main finding is that 3 months after the acute event patients affected by subcortical stroke presented a reduction in contralateral SP duration in the unaffected hemisphere; this trend is related to clinical improvement of upper limb motor function. In conclusion, SP proved to be a valid parameter to characterize cortical reorganization patterns in stroke survivors and provided useful information about motor recovery within 3 months in subcortical patients.

## Introduction

New frontiers in stroke rehabilitation aim to improve functional recovery taking advantage from the knowledge of mechanisms of cortical reorganization that occur after the acute event (Schaechter, 2004), the so-called “top-down” approach (Chisari, 2015). Literature findings have provided insight into the mechanisms of behavioral rehabilitation techniques, such as constraint-induced movement therapy (Liepert, 2006), and have led to the development of cortical stimulation protocols to improve upper limb recovery (Ward and Cohen, 2004; Nowak et al., 2008; Chisari et al., 2014). The main hypothesis is that an imbalanced inter-hemispheric inhibition occurs following stroke, so the purpose of various rehabilitation approaches is to increase excitability of perilesional intact regions of the affected hemisphere and/or to decrease excitability of the contralesional hemisphere (Hummel and Cohen, 2006; Nowak et al., 2009). Anyway, until now still no customized treatment has been proposed strictly based on the correlations between neurophysiological and functional evaluations.

Transcranial magnetic stimulation (TMS) is a valid tool to obtain data about cortical reorganization (Rossini et al., 2003). TMS is currently used to elicit motor evoked potential (MEP), recorded by surface electromyography (EMG). MEP presence, as a measure of cortical excitability changes and corticospinal tract integrity, offers useful prognostic information about functional outcome (Hendricks et al., 2002; Brouwer and Schryburt-Brown, 2006; Pizzi et al., 2009; Stinear et al., 2014). In pre-activated muscles, TMS may also induce a transient suppression of the EMG-activity after MEP, the so-called silent period (SP) (Kukowski and Haug, 1991; Uozumi et al., 1992), as an inhibitory effect. SP is reported to be abnormally increased in the paretic hand after a stroke (Haug and Kukowski, 1994; Braune and Fritz, 1996; Harris-Love et al., 2016) and tend to decrease with motor recovery. To date, studies on the role of the SP in predicting motor recovery after severe stroke showed rather inconsistent results (van Kuijk et al., 2005, 2014).

Starting from the study conducted by Liepert et al. (2005) the impact of lesion location on motor excitability and motor performance was investigated. The authors evaluated patients with pure motor strokes in four different brain areas (motor cortex lesions, striatocapsular lesions, lacunar lesions of the internal capsule and paramedian pontine lesions), concluding that lesion location determines a specific pattern of motor excitability changes. Recently Thickbroom et al. (2015) highlighted that both the anatomical level of the lesion and to the degree of paretic motor impairment are related cortical excitability and reorganization after stroke. These findings suggest that rehabilitative trials should stratify patients basing on lesion type.

Coupar et al. (2011) also suggest that integrating early clinical data with neurophysiological measurements could be useful to predict long-term recovery and outcome. A remarkable example is the study conducted by Di Lazzaro et al. (2010), which evaluated whether long-term potentiation (LTP)- and long-term depression (LTD)-like changes produced by intermittent theta burst stimulation (iTBS) in acute stroke correlate with outcome at 6 months. They recruited ischemic stroke patients (both with cortical and subcortical lesions) in the first 10 days after stroke, finding that functional recovery is directly correlated with LTP-like changes in affected hemisphere (AH) and LTD-like changes in unaffected hemisphere (UH) and inversely correlated with the baseline excitability of UH. Nevertheless, it is suitable to underline that neurophysiological data during early period post-stroke may suffer from wide inter-subject variability. In particular, Swayne et al. (2008) found that day-to-day variation in clinical performance was unrelated to physiological measures in the first days after the acute event.

Following these considerations our hypothesis was that different stroke lesion location may imply differences in the mechanisms of brain reorganization that lead motor recovery in subacute phase. Our aim was to identify neurophysiological parameters that can be used as markers to describe motor recovery and as factors to guide neurorehabilitation treatment in subacute stroke patients with different lesions.

For this reason we correlated neurophysiological and functional features in a cohort of stroke patients recruited in a specific time window from the acute event and subdivided in cortico-subcortical and subcortical strokes; evaluations were also performed at 3 months after stroke to monitor changes in brain reorganization and clinical behavior.

## Materials and methods

### Participants and study protocol

A total of 82 stroke patients were screened at the Neurorehabilitation Unit of University Hospital of Pisa. Inclusion criteria were: (1) age ranged between 18 and 80 years; (2) first-ever unilateral ischemic stroke; (3) time from acute event within 45 days. Exclusion criteria were: (1) TMS contraindications (cardiac pacemaker, use of drugs targeting CNS, diagnosis of epilepsy); (2) MMSE <24.

Twenty subjects were excluded for the hemorrhagic nature of the event and another 20 because they have previously experienced a cerebrovascular event. Among the 42 remaining patients, other 12 were excluded because they met our exclusion criteria.

Therefore, 30 ischemic stroke patients (M/F: 19/11; mean age ± SD: 69.51 ± 13.59 years) were enrolled and divided in two groups according to lesion site: 15 with cortical-subcortical (CS) and 15 with subcortical (S) lesion. Finally, 20 of them ended all the evaluations (M/F: 10/10; 9 with CS and 11 with S lesion; 7 with right lesioned hemisphere and 13 with left lesions). Based on brain CT images, lesions were defined as “subcortical” if they involved the deep white matter inferior to the corpus callosum, including the internal capsule, thalamus and basal ganglia and spared the cerebral cortex. Otherwise all lesions including also a cortical involvement were defined a “cortico-subcortical.”

Data about age, gender, affected hemisphere (right or left), lesion location, time since stroke and drugs used were obtained from all subjects. Fifteen healthy right-handed control subjects (M/F: 9/6; mean age ± SD: 33.9 ± 14.75 years), were also included. They did not present any neurological or other severe medical diseases. They did not have contraindications for TMS.

Clinical and neurophysiological assessments were performed at baseline, in a range of 10–45 days (t0), and at 3 months (t1) after the acute event.

Each patient and healthy subject recruited gave their written informed consent. This study was authorized by local Ethics committee of Area Vasta Nord Ovest (CEAVNO) for Clinical experimentation, Tuscany (Italy).

### Transcranial magnetic stimulation

TMS was performed using a MagProX100 MagOption stimulator (MagVenture, Farum, Denmark) connected to a figure-eight coil. During stimulation, the coil was held with the handle pointing postero-laterally at an angle of 45° to midline. Participants were comfortably seated in a chair with forearms and hand supinated and supported by a custom built device. The elbow was positioned in 90° flexion. Surface EMG recordings were made bilaterally from the abductor pollicis brevis muscle (APB). The position at which the stimulation produced optimal MEP in the contralateral APB was identified on each side. The raw EMG signal acquired at 512 Hz sampling frequency, was then amplified and band-pass filtered (30–1000 Hz) by Neurotravel Win—Cadwell (Ates Medical Device) using Sierra Wave software. The off-line analysis was made on a laboratory computer with custom routines written in Matlab (The MathWorks Inc., Natick, MA, USA).

Resting Motor Threshold (RMT) and contralateral SP (cSP) duration parameters were collected from both hemisphere in stroke patients and from dominant hemisphere in control subjects. RMT was defined as the intensity needed to elicit a response with >50 mV amplitude (peak-to-peak) in at least 5 of 10 stimuli. RMT value was defined as 100% if no MEP could be evoked at maximum stimulator output (MSO) (Takechi et al., 2014). SP was measured applying a TMS pulse at a suprathreshold intensity of 120% of RMT when the APB muscle was actively contracted. The cSP duration was defined between the MEP onset and the return of continuous EMG-activity to pre-stimulus levels (van Kuijk et al., 2014). TMS was delivered 100 ms after starting EMG recording with a 500 ms storage sweep so that pre- and post-stimulus activity could be satisfactorily recorded. TMS pulses were delivered during voluntary isometric muscle contraction of approximately 5 s with the instruction to maintain contraction at least 2 s after the stimulus. The contraction force was set to 20% of maximal force level with online monitoring.

Five cSP trials for each hemisphere in each patient were collected and then analyzed after getting the mean value. Furthermore, background EMG levels were determined with the root mean square (RMS) amplitude of the EMG activity during the time period from 100 to 25 ms before TMS stimulus delivery.

### Clinical evaluations

Clinical scales to evaluate activities of daily living (Barthel Index—BI) and upper limb motor function (Fugl-Meyer Assessment for the upper limb—FMA-UL and Wolf Motor Function Test—WMFT) were also administered. BI is an assessment scale divided in 10 items to evaluate patient's ability to care for him/herself (Mahoney and Barthel, 1965). FMA is a feasible, well-designed and widely used scale for global clinical examination. FMA for the upper limb can be divided in 4 items: sensory function, pain, passive joint motion and motor function (Fugl-Meyer et al., 1974; Gladstone et al., 2002). WMFT is a time-based method to evaluate upper extremity function while providing insight into joint-specific and total limb movements (Wolf et al., 2001).

### Data analysis and statistics

Our sample was characterized by a non-Gaussian distribution (Shapiro-Wilk's *P* > 0.01) then non-parametric statistics was used. Wilcoxon signed-rank test was used to analyse longitudinal changes between t0 and t1 in CS and S subgroups. Clinical and neurophysiological data were compared between the two stroke subgroups and neurophysiological data were also compared between the whole stroke group and controls. Clinical data were also analyzed in other two subgroups identified by the presence of MEP at t0. The difference in the same value between t0 and t1 was calculated subtracting from the t1 value the t0 value (Δ). Besides, we calculated the percentage of increase or decrease of time parameters such as WMFT time (indicated with %). Outlier rejection was made using a calculator performing Grubbs' test. Spearman's Correlation Coefficient was used to identify possible correlation between TMS variables and clinical scores at two different time points. Furthermore, to assess differences between study groups (CS and S or MEP±) Mann-Whitney's U test was used. Bonferroni correction was applied for multiple comparisons.

To rule out systematic differences in background EMG over time or within groups that could impact on SP duration, we also performed repeated measures ANOVA analysis of RMS values.

IBM SPSS Statistics 20 software for analysis was used.

## Results

### Clinical findings

**Figure 1:**
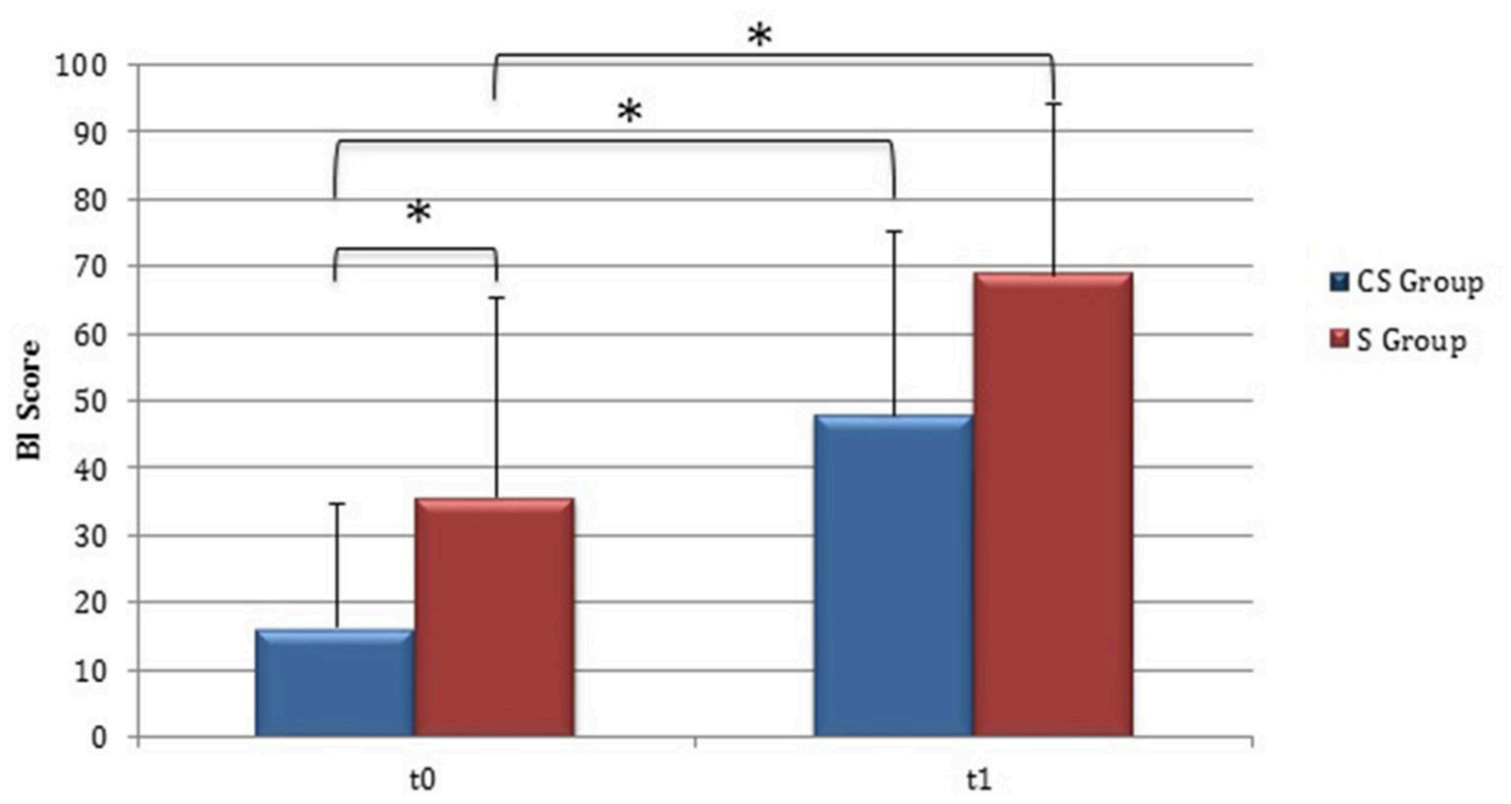
**Barthel Index (at baseline and at 3 months)**. Barthel Index score at baseline and at t1. The blue histogram corresponds to cortico-subcortical lesioned patients, the pink one to subcortical stroke patients. BI, Barthel Index; CS, cortico-subcortical stroke patients; S, subcortical stroke patients. ^*^P < 0.05.

Clinical evaluations were administered at baseline (Table 1) and at t1 to all patients (Table 2). At baseline, no statistically significant differences were observed between two subgroups except for Barthel Index, in which S patients demonstrated a significant better score compared with CS patients (*P* = 0.023) (Figure 1).

**Table 1:** **Patient characteristics and clinical evaluations at baseline**.

**Patient**	**Age**	**Gender**	**Lesion site**	**Affected Arm**	**t0 (days)**	**BI**	**FMA-UL MF**	**FMA–UL PjM**	**FMA–UL P**	**FMA–UL SF**	**FMA–UL Tot**	**WMFT score**	**WMFT time (sec.)**
1	54	M	CS	L	31	5	6	22	22	7	57	6	1451
2	65	M	CS	L	15	5	5	22	20	2	49	4	1568
3	79	F	CS	R	23	30	56	24	24	NV	104	65	70
4	85	M	CS	R	45	0	2	21	24	NV	47	0	1800
5	76	M	CS	R	26	25	4	20	24	11	59	0	1800
6	80	F	CS	R	45	5	18	22	24	6	70	19	1050
7	77	F	CS	L	14	10	4	24	24	6	58	0	1800
8	59	M	CS	L	19	90	51	24	23	11	109	66	98.21
9	73	F	CS	L	34	10	45	19	24	9	97	50	120
10	61	F	CS	R	14	85	62	24	24	7	94	74	52.91
11	54	M	S	L	22	35	17	23	21	9	70	38	370
12	59	M	S	R	45	10	8	22	20	NV	50	0	1800
13	78	M	S	R	19	30	52	24	20	12	108	74	52
14	63	M	S	L	19	30	19	22	24	12	77	16	897
15	65	M	S	R	32	35	21	21	23	12	77	29	482
16	22	M	S	R	21	95	62	24	24	12	122	66	39
17	82	F	S	L	27	20	48	22	24	12	106	58	104
18	52	M	S	R	15	100	65	24	24	12	125	67	38
19	73	M	S	L	11	55	40	24	22	11	97	60	64
20	80	M	S	L	18	20	9	23	24	11	65	4	1457

*General characteristics of each patient: age, gender, lesion location (Cortico-subcortical, CS, or purely subcortical, S), affected arm and number of days between the acute event and baseline evaluation (T0). Clinical assessment: BI (Barthel Index); FMA -UL (Fugl-Meyer Assessment for upper limb motor function) Total score (Tot) and sub scores of Motor function (MF), Passive joint motion (PJM), Pain (P), and Sensory function (SF); WMFT (Wolf Motor Function Test) Score and Time*.

**Table 2:** **Patients' clinical evaluations at baseline and at t1**.

**Patient**	**BI (t0)**	**BI (t1)**	**ΔBI**	**FMA-UL (t0)**	**FMA-UL PJM (t0)**	**FMA-UL P (t0)**	**FMA–UL SF (t0)**	**FMA–UL Tot (t0)**	**FMA–UL MF (t1)**	**FMA–UL PJM (t1)**	**FMA–UL P (t1)**	**FMA–UL SF (t1)**	**FMA–UL Tot (t1)**	**ΔFMA–UL Tot**	**WMFT Score (t0)**	**WMFT Time (sec.) (t0)**	**WMFT Score (t1)**	**WMFT Time (sec.) (t1)**	**ΔWMFT Score**	**ΔWMFT Time (sec.)**
1	5	30	25	6	22	22	7	57	6	24	23	8	61	4	6	1451	6	1403	0	−48
2	5	35	30	5	22	20	2	49	8	22	17	8	55	6	4	1568	4	1547	0	−21
3	0	30	30	56	24	24	0	104	63	24	24	0	111	7	65	70	73	60	8	−10
4	0	15	15	2	21	24	NV	47	4	21	24	NV	49	3	0	1800	0	1800	0	0
5	25	60	35	4	20	24	11	59	9	20	22	11	64	5	0	1800	3	1684.5	3	−115.5
6	5	30	25	18	22	24	6	70	23	22	24	6	75	5	19	1050	22	1022	3	−28
7	10	20	10	4	24	24	6	58	6	24	24	6	60	2	0	1800	0	1800	0	0
8	90	100	10	51	24	23	11	109	59	24	22	11	117	8	66	98.21	73	74.04	7	−24.17
9	10	55	45	45	19	24	9	97	65	24	23	10	122	25	50	120	73	74	23	−46
10	85	85	0	62	24	24	7	94	62	24	24	7	94	0	74	52.91	75	48.26	1	−4.65
11	35	65	30	17	23	21	9	70	35	23	21	9	88	18	38	370	47	314	9	−56
12	10	20	10	8	22	20	0	50	8	23	22	0	53	3	0	1800	0	1800	0	0
13	30	75	45	52	24	20	12	108	65	24	23	12	124	16	74	52	75	39	1	−13
14	30	55	25	19	22	24	12	77	35	24	19	10	88	11	16	897	52	124	36	−773
15	35	55	20	21	21	23	12	77	36	24	24	12	96	19	29	482	40	269	11	−213
16	95	100	5	62	24	24	12	122	66	24	24	12	126	4	66	39	75	16	9	−23
17	20	20	0	48	22	24	12	106	60	24	24	12	120	14	58	104	68	38	10	−66
18	100	100	0	65	24	24	12	125	66	24	24	12	126	1	67	38	75	22	8	−16
19	55	75	20	40	24	22	11	97	64	24	24	12	124	27	60	64	70	34	10	−30
20	20	50	30	9	23	24	11	65	31	23	21	12	87	22	4	1457	28	706	24	−751

*Clinical assessment: BI, (Barthel Index); FMA -UL (Fugl-Meyer Assessment for upper limb motor function) Total score (Tot) and sub scores of Motor function (MF), Passive joint motion (PJM), Pain (P), and Sensory function (SF); WMFT (Wolf Motor Function Test) Score and Time. Δ values (difference between t1 and t0) for BI, FMA-UL Tot, and WMFT Score and Time are also reported*.

**Figure 2:**
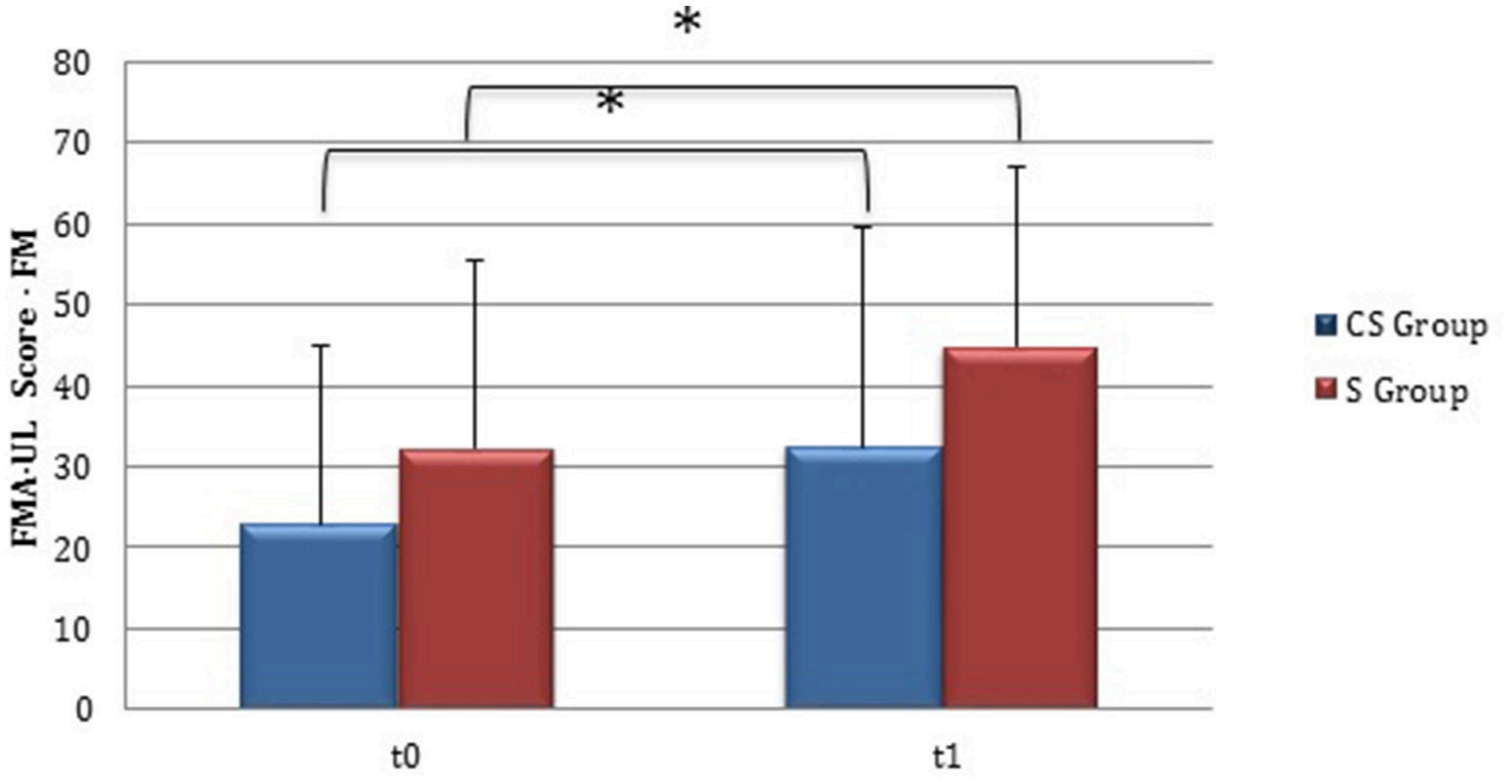
**Fugl-Meyer Assessment for the upper limb—motor function (at baseline and at 3 months)**. Fugl-Meyer Assessment for the upper limb—Motor function subscore values at baseline and at t1. The blue histogram corresponds to cortico-subcortical lesioned patients, the pink one to subcortical stroke patients. FMA-UL, Fugl-Meyer Assessment for the upper limb; FM, motor function subscore; CS, cortico-subcortical stroke patients; S, subcortical stroke patients. ^*^P < 0.05.

**Figure 3:**
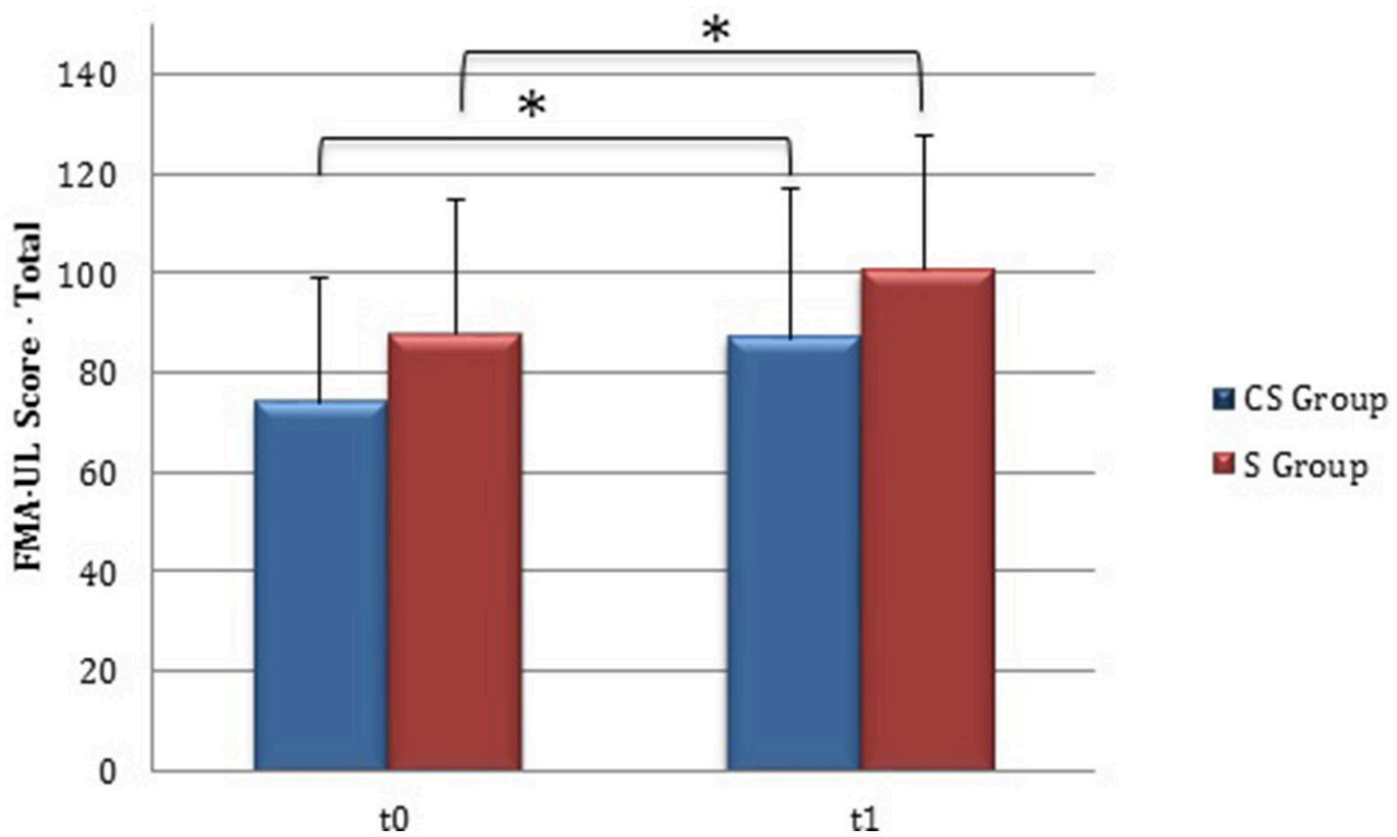
**Fugl-Meyer Assessment for the upper limb—total score (at baseline and at 3 months)**. Fugl-Meyer Assessment for the upper limb—total score values at baseline and at t1. The blue histogram corresponds to cortico-subcortical lesioned patients, the pink one to subcortical stroke patients. FMA-UL, Fugl-Meyer Assessment for the upper limb; CS, cortico-subcortical stroke patients; S, subcortical stroke patients. ^*^P < 0.05.

**Figure 4:**
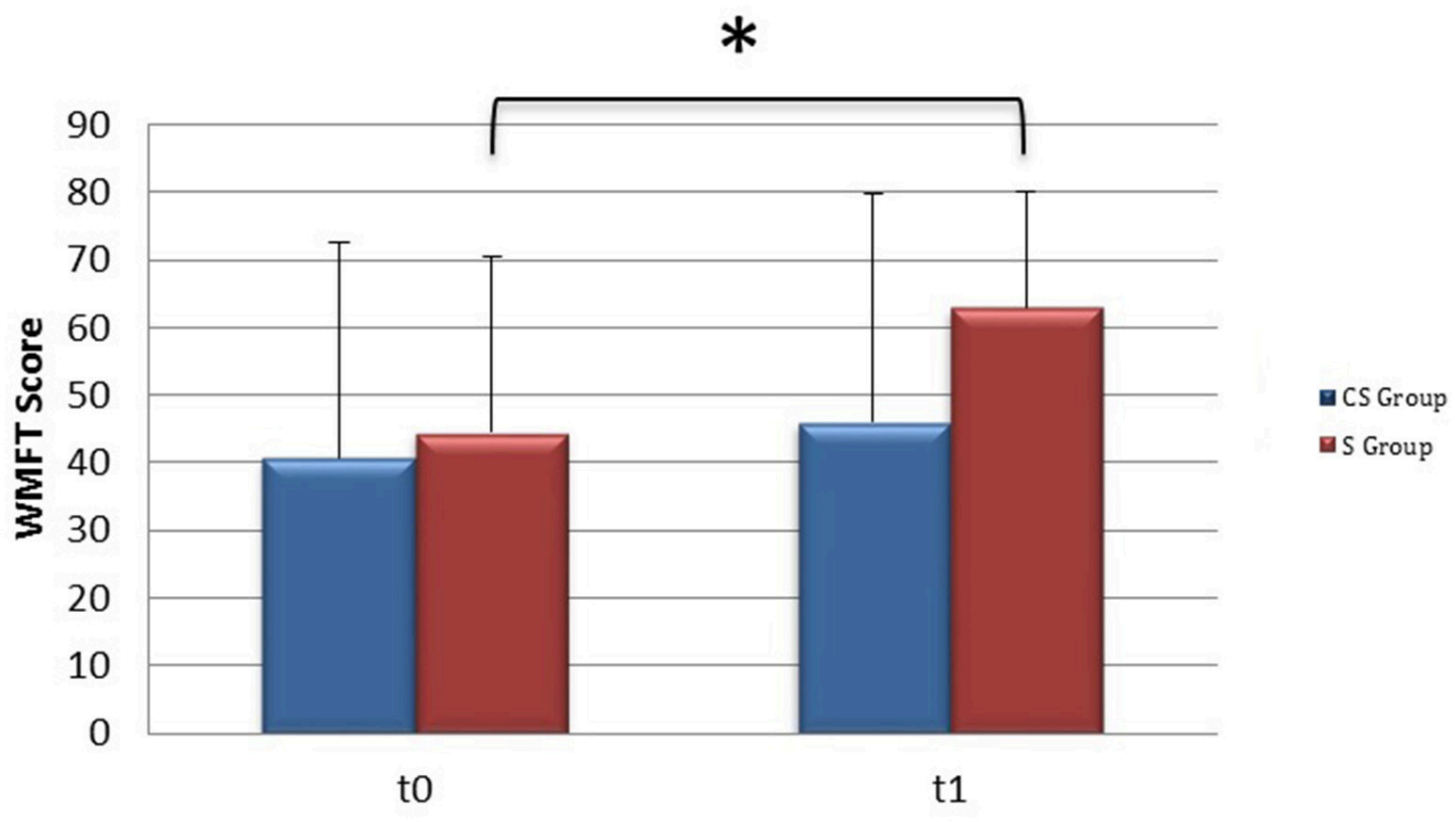
**Wolf Motor Function Test score (at baseline and at 3 months)**. Wolf Motor Function Test Score at baseline and at t1. The blue histogram corresponds to cortico-subcortical lesioned patients, the pink one to subcortical stroke patients. WMFT, Wolf Motor Function Test; CS, cortico-subcortical stroke patients; S, subcortical stroke patients. ^*^P < 0.05.

**Figure 5:**
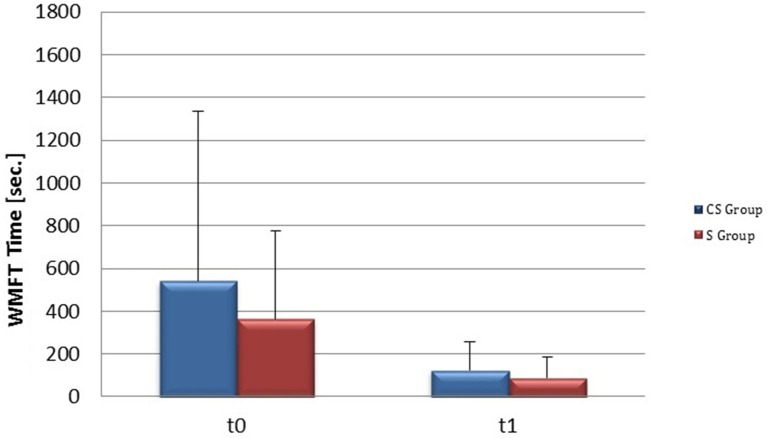
**Wolf Motor Function Test time (at baseline and at 3 months)**. Wolf Motor Function Test time at baseline and at t1. The blue histogram corresponds to cortico-subcortical lesioned patients, the pink one to subcortical stroke patients. WMFT, Wolf Motor Function Test; CS, cortico-subcortical stroke patients; S, subcortical stroke patients.

From baseline to t1, significant functional improvement was evident in BI in CS and S groups (*P* = 0.008 and *P* = 0.012, respectively) (Figure 1), in FMA-UL motor function subscore (*P* = 0.012 and *P* = 0.008, respectively) (Figure 2), in FMA-UL total score (*P* = 0.008 and *P* = 0.005, respectively) (Figure 3). However, only S group showed a significant improvement in upper limb fine motility, expressed in WMFT score (*P* = 0.008) (Figure 4) and time (*P* = 0.008) (Figure 5), while improvement in fine motility scale observed in CS group was marginally significant [WMFT score (*P* = 0.027) and time (*P* = 0.028) (Figures 4, 5)].

### Neurophysiological data

#### Motor evoked potentials

**Figure 6:**
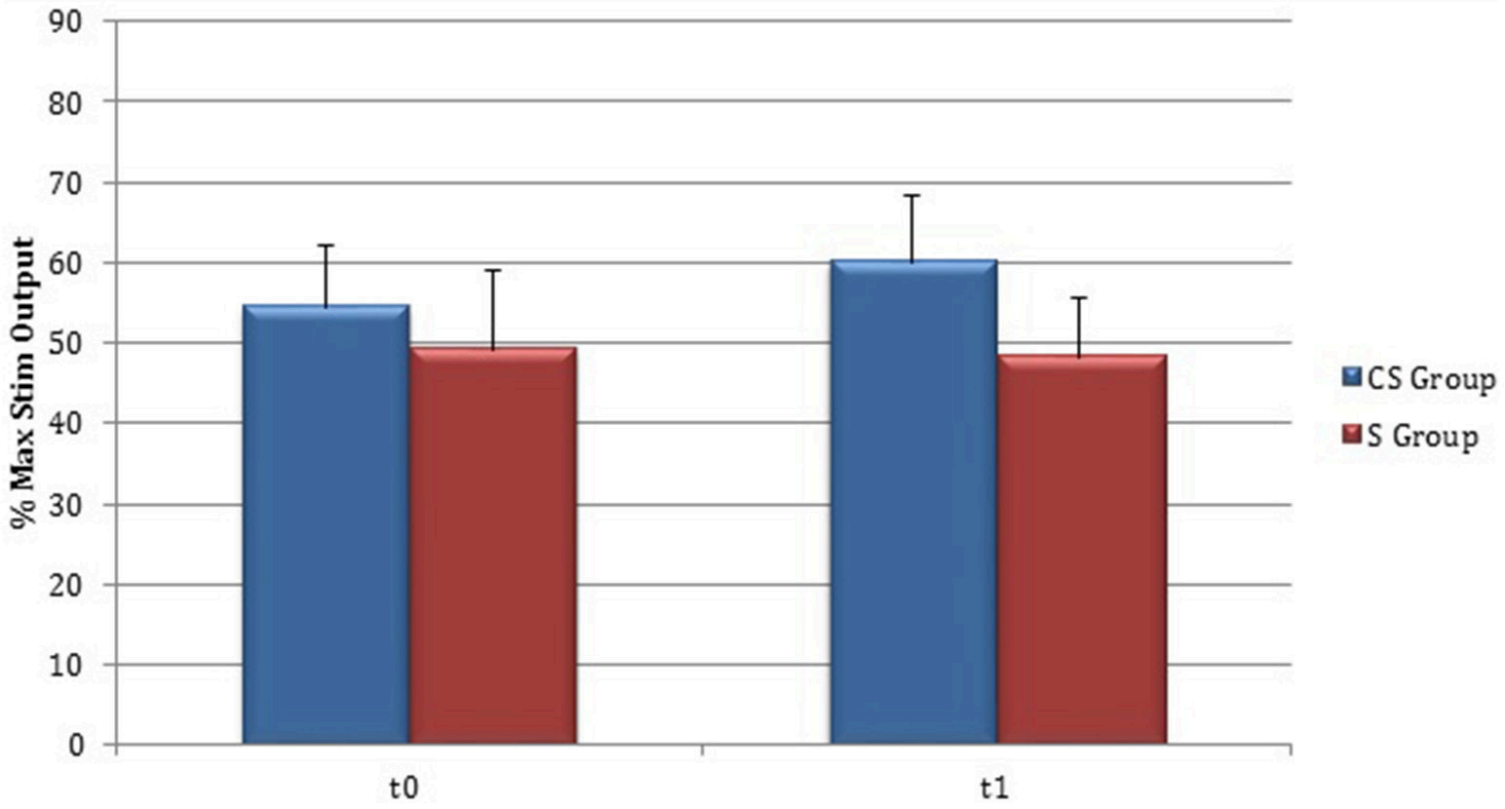
**Motor evoked potentials: Resting Motor Threshold in the unaffected hemisphere**. The figure represents Resting Motor Threshold (RMT) for stimulation of unaffected hemisphere at t0 and t1. RMT value was described as 100% if no MEP could be evoked at maximum stimulator output. CS, cortico-subcortical stroke patients; S, subcortical stroke patients.

**Figure 7:**
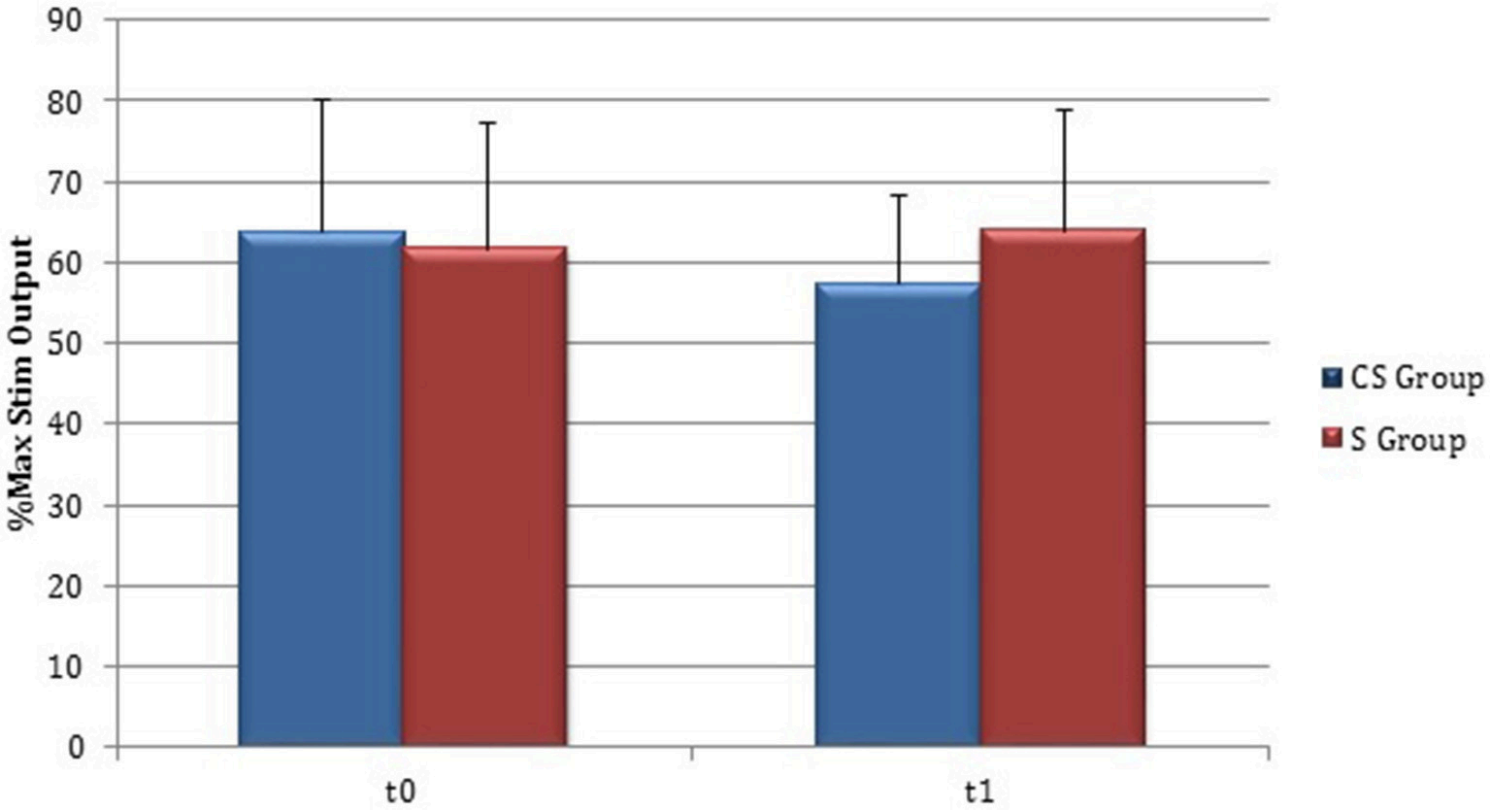
**Motor evoked potentials: Resting Motor Threshold in the affected hemisphere**. The figure represents Resting Motor Threshold (RMT) for stimulation of affected hemisphere at t0 and t1. RMT value was described as 100% if no MEP could be evoked at maximum stimulator output. CS, cortico-subcortical stroke patients; S, subcortical stroke patients.

RMT was recorded from both hemisphere in all stroke patients and from the dominant side in controls (Table 3). A wide inter-subject variability in RMT values was evident. In particular, analysis at t0 did not show a significant difference between the mean value of RMT in stroke patients and controls (UH *P* = 0.797; AH *P* = 0.152). Moreover, at t0 no significant difference in RMT values was found comparing the two stroke subgroups (UH *P* = 0.127; AH *P* = 0.794) (Figures 6, 7).

**Table 3:** **Neurophysiological parameters: motor evoked potentials and cortical silent period**.

	**Whole stroke group**	**CS group**	**S group**	**Controls**
UH RMT t0	51.6315789	54.4444444	49.1	54.6428571
UH RMT t1	54.15	60	48.3	
AH RMT t0	62.5714286	63.8333333	61.625	54.6428571
AH RMT t1	61.3333333	57.5	63.8888889	
UH cSP t0	0.14880298	0.1334	0.16035521	0.13915
UH cSP t1	0.14627556	0.17294762	0.1229375	
AH cSP t0	0.20652727	0.169275	0.22781429	0.13915
AH cSP t1	0.17679679	0.182405	0.17329167	

*UH, Unaffected Hemisphere; AH, Affected Hemisphere; RMT, Resting Motor Threshold; cSP, contralateral Silent Period; CS, cortico-subcortical stroke patients; S, subcortical stroke patients*.

Patients in which MEPs could not be elicited (and RMT was defined as 100% of MSO) were: three at t0 for cortico-subcortical group and two at t1; for subcortical group two patients at t0 and one at t1.

#### Silent period

As regards Silent Period (SP) recordings, cSP was collected from both hemispheres in all stroke subjects and from dominant hemisphere in controls (Table 3).

**Figure 8:**
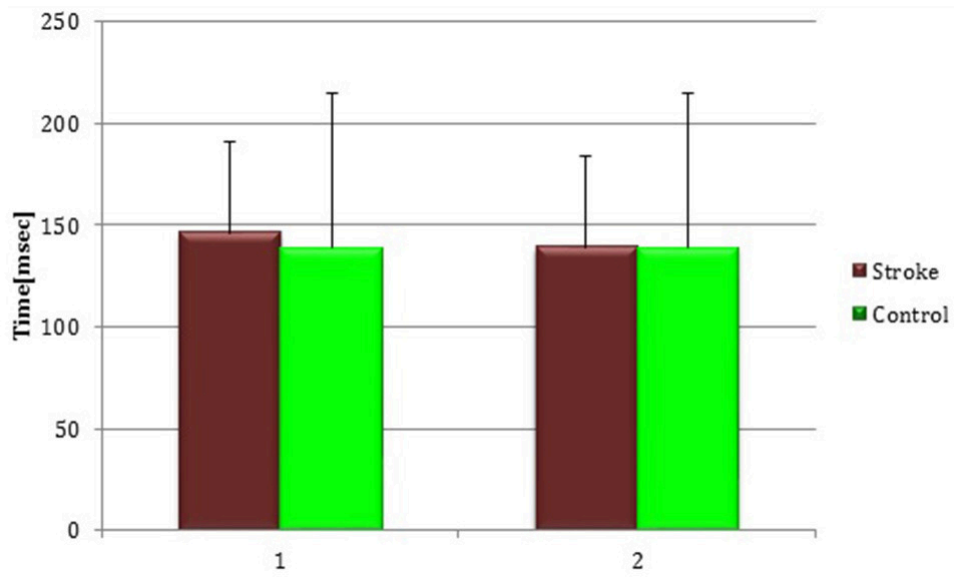
**Cortical Silent Period in the whole stroke group and in controls**. The figure represents contralateral Silent Period recorded by unaffected hemisphere stimulation of whole stroke group and dominant hemisphere in controls. Silent Period were collected at t0 (1) and t1 (2) in stroke patients, while control subjects were evaluated only once (therefore column 1 and 2 are equal for this group).

**Figure 9:**
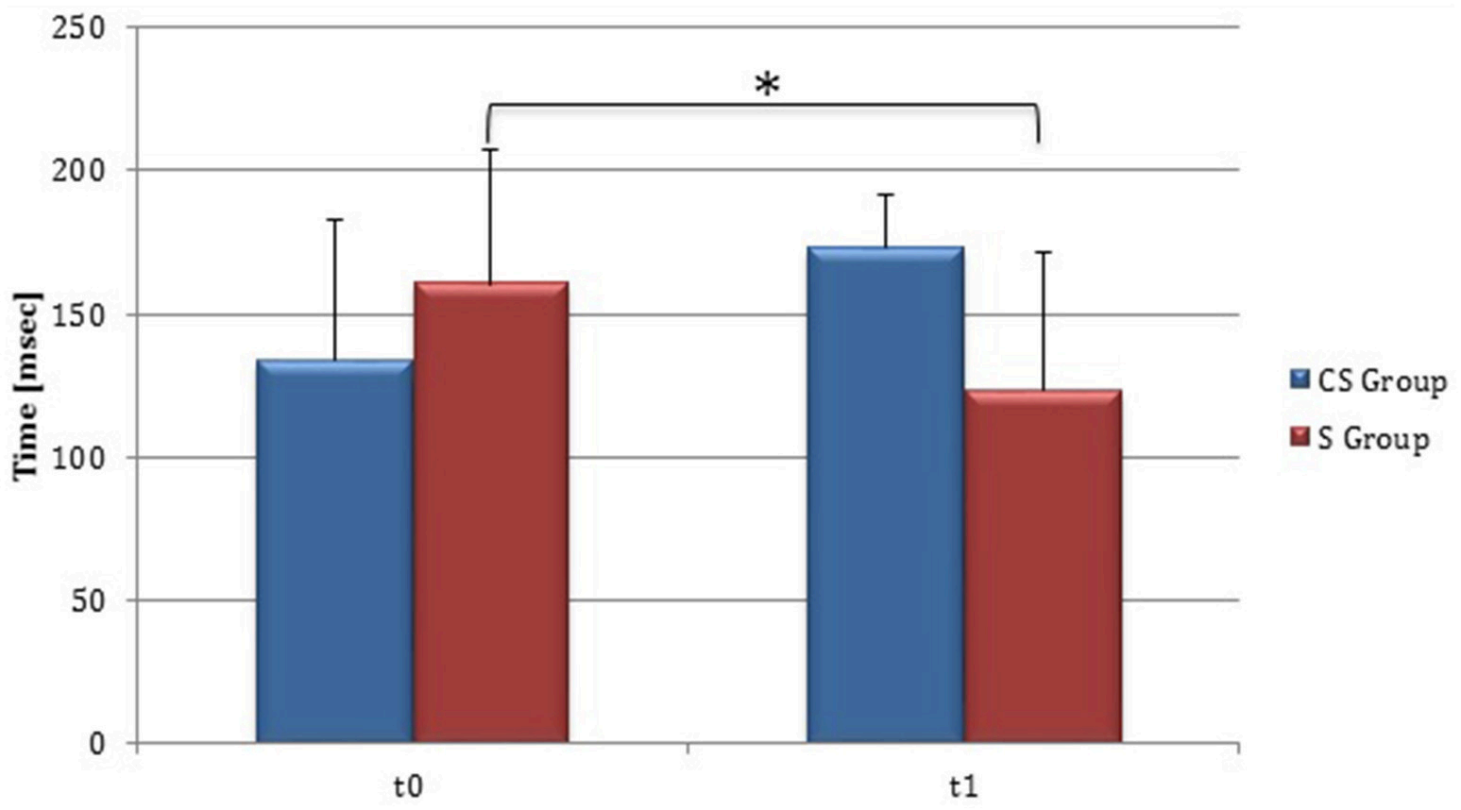
**Cortical Silent Period in the unaffected hemisphere**. The figure represents contralateral Silent Period recorded by unaffected hemisphere stimulation at t0 and t1 in cortico-subcortical and subcortical stroke patients. CS, cortico-subcortical stroke patients; S, subcortical stroke patients. ^*^P < 0.05.

At baseline no significant difference was found by comparing the whole stroke group with controls (*P* = 0.710) (Figure 8). From t0 to t1 a statistically significant reduction in UH cSP in S patients was found (*P* = 0.049) (Figure 9).

**Figure 10:**
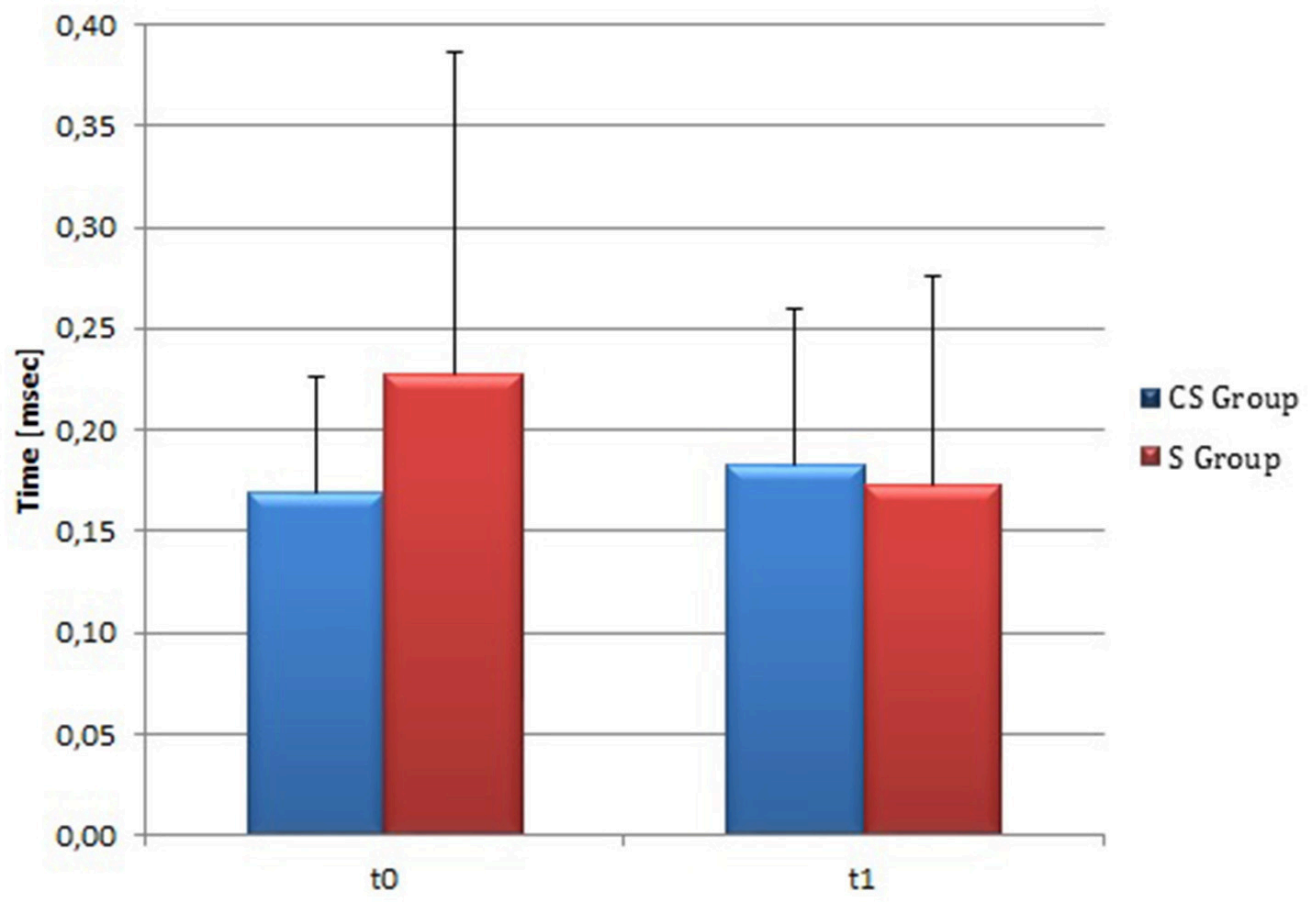
**Cortical Silent Period in the affected hemisphere**. The figure represents contralateral Silent Period recorded by affected hemisphere stimulation at t0 and t1 in cortico-subcortical and subcortical stroke patients. CS, cortico-subcortical stroke patients; S, subcortical stroke patients.

At t0 no statistically significant difference was found in cSP recorded from AH between the two stroke subgroups (*P* = 0.571). AH cSP decreased at t1 in S group, but this trend was not statistically significant (*P* = 0.463) (Figure 10).

RMS analysis over time and within groups showed that the differences in background EMG levels did not impact on the interpretation of silent period data in our sample.

### Correlations between clinical and neurophysiological parameters

The relationship between clinical and neurophysiological parameters was also explored. In particular we analyzed time-related parameters' changes from t0 to t1.

**Figure 11:**
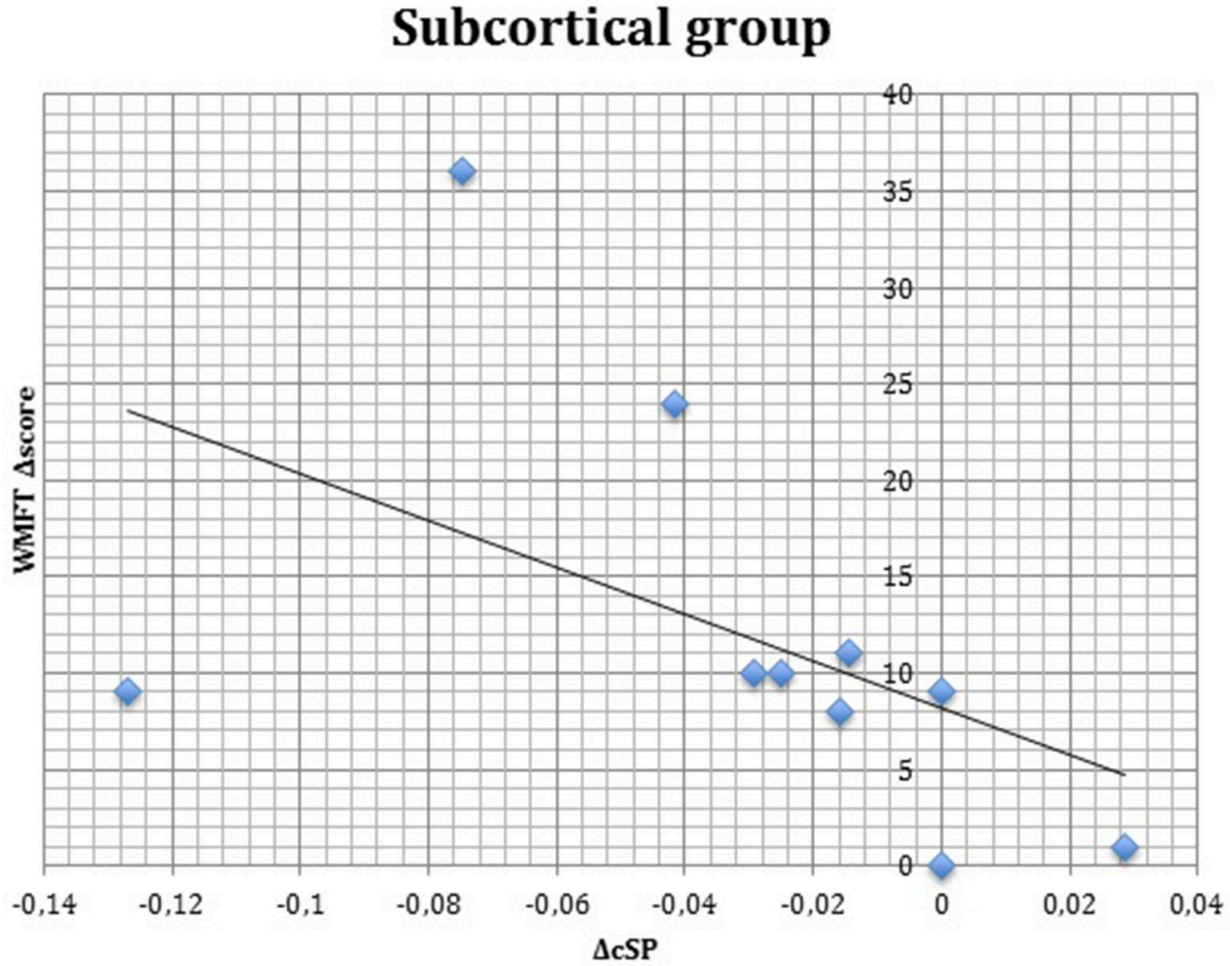
**Correlations between clinical and neurophysiological parameters**. Correlation between ΔcSP on unaffected hemisphere and ΔWMFT score in subcortical stroke subgroup. cSP, contralateral Silent Period; WMFT, Wolf Motor Function Test; Δ, The difference between t0 and t1 in the same value, calculated subtracting as t1–t0.

**Figure 12:**
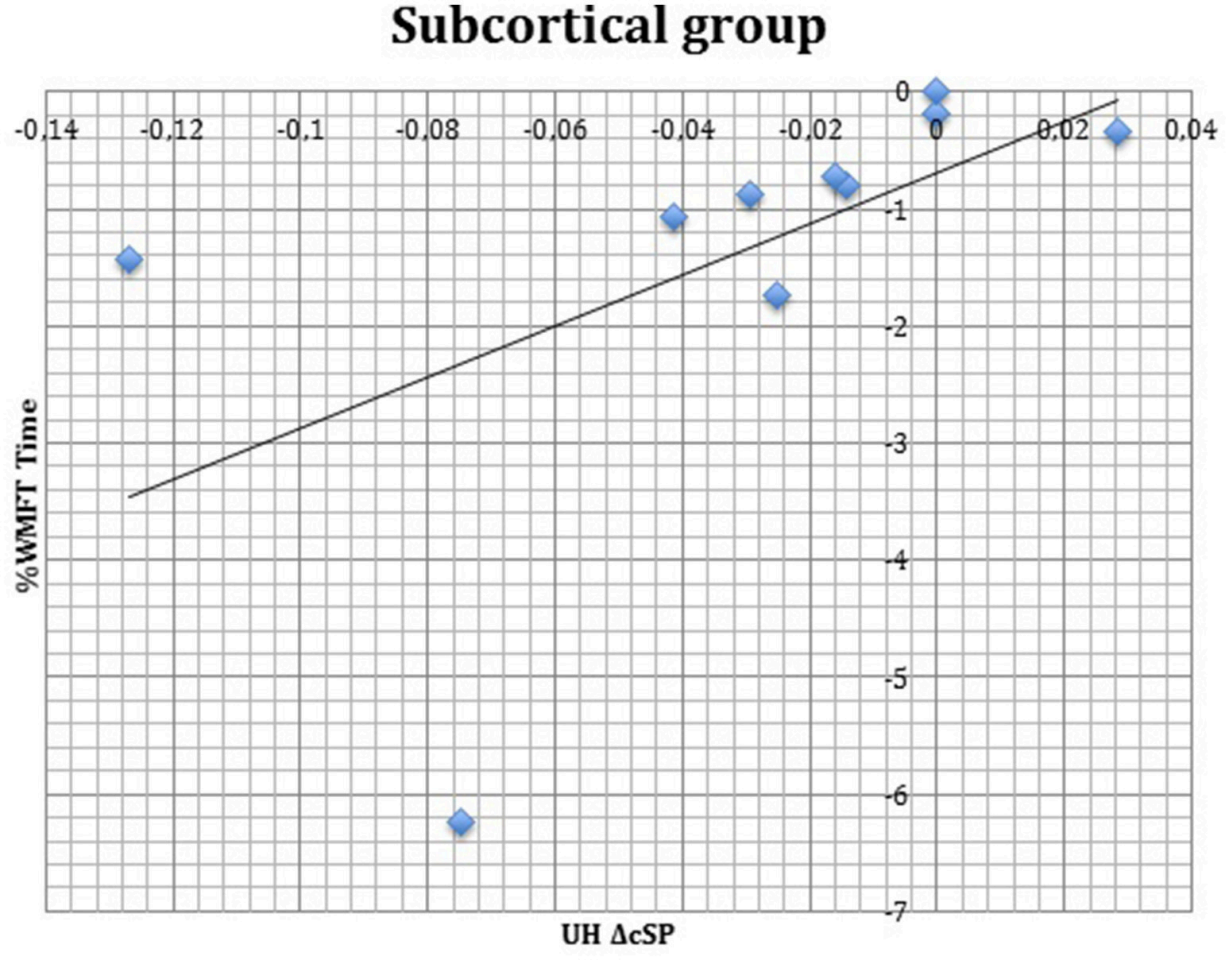
**Correlations between clinical and neurophysiological parameters**. Correlation between ΔcSP on UH and %WMFT time in subcortical stroke subgroup. cSP, contralateral Silent Period; WMFT, Wolf Motor Function Test; UH, unaffected hemisphere; %, the percentage of increase or decrease of WMFT time.

S group showed a negative correlation between ΔcSP on UH and ΔWMFT score (*P* = 0.049; Spearman's Rho = −0.633) (Figure 11) and a positive correlation with %WMFT time (*P* = 0.002; Spearman's Rho = 0.851) (Figure 12). It means that the reduction of cSP on UH was paralleled by an increase on WMFT score and a decrease of WMFT time. Correlations analysis in S group showed that ΔcSP on AH negatively related with FMA-UL—passive motility subscore at t0 (*P* = 0.044; Spearman's Rho = −0.819), FMA-UL—motor function subscore at t0 (*P* = 0.032; Spearman's Rho = −0.675), FMA-UL—total score at t0 (*P* = 0.034 Spearman's Rho = −0.671), WMFT score at t0 (*P* = 0.009; Spearman's Rho = −0.772). Moreover, the reduction of AH cSP from t0 to t1 (ΔcSP) in S patients, reflected a higher score of BI (*P* = 0.048; Spearman's Rho = −0.637), FMA-UL—motor function subscore (*P* = 0.012; Spearman's Rho = −0.755), FMA-UL—total score (*P* = 0.010; Spearman's Rho = −0.764), WMFT score (*P* = 0.012; Spearman's Rho = −0.751), at t1. In the same stroke subgroup ΔcSP on AH positively related with WMFT time at t0 (*P* = 0.007; Spearman's Rho = 0.784) and at t1 (*P* = 0.018; Spearman's Rho = 0.723) and negatively related with ΔWMFT time (*P* = 0.039; Spearman's Rho = −0.657): the reduction of cSP values on AH corresponds to shorter times of execution in WMFT.

#### Clinical scales in groups divided for MEP presence

**Figure 13:**
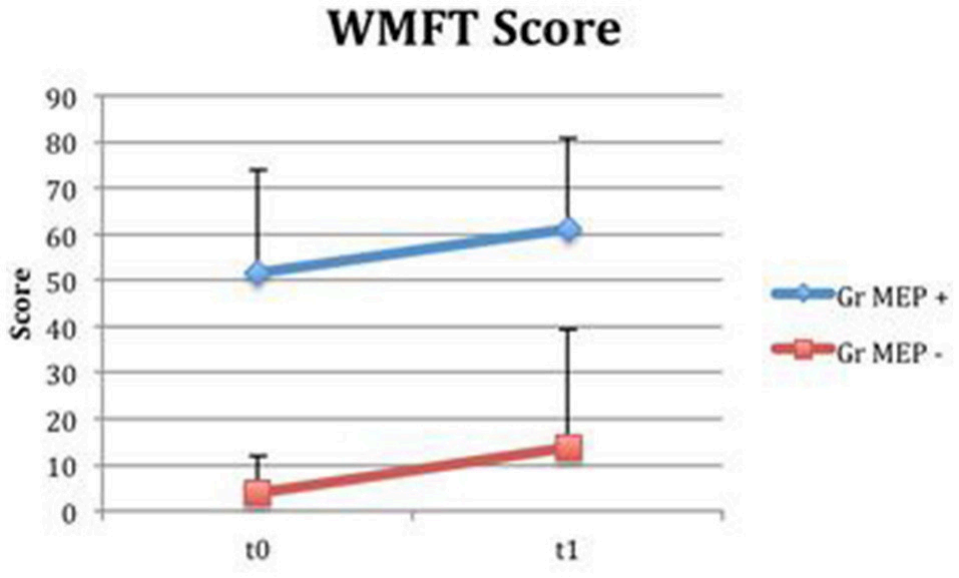
**Wolf Motor Function Test Score in groups divided for MEP presence**. Fine hand mobility improvement (assessed with WMFT) in groups divided based on MEP presence (MEP +, represented in blue) or absence (MEP –, represented in red) at t0.

Even if both groups presented a time-related improvement, the group with MEP at t0 presented higher fine hand mobility values than the group without MEP at t0, assessed with WMFTs (Figure 13) and WMFT t both at baseline (*P* = 0.004; *P* = 0.006) and at t1 (*P* = 0.012; *P* = 0.013). Improvements in the two groups (measures by Δ) were not statistically different.

## Discussion

Our main finding is that the group of patients with subcortical stroke evaluated in this study presented a reduction in contralateral Silent Period (SP) duration in the unaffected hemisphere and this trend is related to clinical improvement 3 months after the acute event. It is well known that the SP can be divided in two parts: an early part principally due to spinal mechanisms and the late part due to inhibition mechanisms of the motor cortex (Chen et al., 1999) mediated through type B-GABAergic interneurons receptors (Di Pino et al., 2014). Literature reported that SP is prolonged in the acute phase after an injury (Liepert et al., 2000b) and it normalizes with the clinical improvement (Classen et al., 1997). In this study we observed a no significant reduction of cSP duration in the AH at 3 months, but this trend (Δ cSP) correlated with functional recovery. Otherwise, the reduction of unaffected intracortical inhibition in S patients may play an important role in effective motor recovery at 3 months.

Changes in cortical activity after stroke have already been investigated in recent literature. Previous studies (Liepert et al., 2000a; Manganotti et al., 2002; Swayne et al., 2008) reported that RMT values were higher on the AH than on the UH. However, the baseline excitability of the AH seems to be less relevant: Swayne et al. (2008) showed a poor correlation between AH neurophysiological features and clinical behavior at 6 months after stroke. The main hypothesis was that the reorganization of alternative cortical networks was more relevant for motor recovery than the function of the original corticospinal tract spared by the ischemic lesions. The role of UH motor cortex in post-lesional recovery is still debated (Takechi et al., 2014). It was supposed that unaffected hemisphere facilitates control of recovered motor function by operating at a higher-order processing level involved in selection, preparation, temporal or spatial organization of movement (Gerloff et al., 2006). Bütefisch et al. (2003) compared stroke patients with good recovery of hand function and controls to determine changes in the inhibitory and excitatory activity in UH motor cortex in stroke patients. They found that in patients' contralesional motor cortex the balance of excitatory and inhibitory activity was shifted toward an increase of excitatory activity; they postulated that in stroke subjects this issue may be relevant for reorganizational processes and may play a role in the functional recovery of patients.

The present study showed substantial stability of RMT values in UH in S group, whereas CS patients showed a no significant increase in RMT value over time. In literature, Manganotti et al. (2002) reported a decrease of RMT in UH detectable in a very early phase after the acute event (5–7 days after stroke) and a trend toward the normalization in patients who experimented a good recovery. Contrarily, the persistence of abnormal excitability in UH was associated with a poor motor recovery. Our data highlighted a substantial stability of UH excitability within 3 months in subcortical patients. The different timing of neurophysiological evaluations may have contributed to mask the effect due to a wider variability. In fact, we described the UH excitability trend in a window between 10 and 45 days after stroke, whereas Manganotti et al. (2002) chose an earlier timing. Swayne et al. (2008) affirmed that the earliest period after stroke is characterized by a wide variability in cortical reorganization and this could account for the different results of our work. Probably, a larger and more homogeneous dataset would help us to resolve this issue and establish a proper timing to characterize neurophysiological changes and correlations with clinical outcome.

Moreover, as already described in literature the presence of MEP in the earliest phase after stroke has a positive predictive value for motor recovery (Stinear et al., 2014). TMS responses were not collected before the first 10 days, so MEP presence at baseline cannot be considered as a predictive factor in our study. However, it emerged that patients with MEP in the affected side at t0 had a better basal clinical score, but not a greater degree of motor recovery.

Regarding the meaning of post-lesional changes observed in the unaffected motor cortex, Shimizu et al. (2002) interpreted the post-lesional cortical changes in the contralateral side as “motor cortex disinhibition,” i.e., a consequent manifestation of the lesion itself. Our data indicate that changes in UH motor cortex could be well described with the modifications detectable with cSP. Moreover, in S patients the cSP demonstrated a clear-cut correlation with clinical outcome.

In conclusion, our analysis proved that cSP is a valid parameter to characterize cortical reorganization patterns in subacute stroke subjects. Furthermore, this measure significantly correlated with motor function in S patients and provided useful information about motor recovery within 3 months.

### Limitations of the study

There are some limitations to consider. First of all, small sample size and variability of the clinical features of patients recruited suggest that results need to be confirmed in further studies, which will include larger samples. Furthermore, control group's mean age was lower than that of stroke patients and it could have influenced results.

As regards baseline evaluations, the first clinical and neurophysiological measures were made between 10 and 45 days post-stroke. No information about the earliest phase were collected. Moreover, changes in both motor performance and neurophysiological measures may occur between 2 and 6 weeks after stroke.

## Author contributions

GL recruited patients, performed clinical and neurophysiological evaluations, contributed to data analysis, interpreted the results and drafted the manuscript; CF performed neurophysiological evaluations, executed data analysis and statistics, helped interpret the results; GS performed clinical and neurophysiological evaluations, contributed to data analysis, helped draft the manuscript; FB performed neurophysiological evaluations, helped to execute data analysis and interpret the results; AP helped perform data analysis and statistics and revised the manuscript; SM was responsible for data analysis and statistics; BR supervised the manuscript; CC designed the study, was responsible for patients' enrolment, contributed to analysis and interpretation of data and revised manuscript. All authors read and approved the final manuscript.

## Funding

The authors disclose receipt of the following financial support for the research, authorship, and/or publication of this article: this work was supported by the Fondazione Pisa (GRANT: 158/2011). The funders had no role in study design, data collection and analysis, decision to publish, or preparation of the manuscript.

### Conflict of interest statement

The authors declare that the research was conducted in the absence of any commercial or financial relationships that could be construed as a potential conflict of interest.

## References

[B1] Braune H.-J. Fritz C. (1996). Asymmetry of silent period evoked by transcranial magnetic stimulation in stroke patients. Acta Neurol. Scand. 93, 168–174. 10.1111/j.1600-0404.1996.tb00194.x 8741138

[B2] Brouwer B. J. Schryburt-Brown K. (2006), Hand function motor cortical output poststroke: are they related? Arch. Phys. Med. Rehabil. 87, 627–634. 10.1016/j.apmr.2006.02.006 16635624

[B3] Bütefisch C. M. Netz J. Webling M. Seitz R. J. Hömberg V. (2003). Remote changes in cortical excitability after stroke. Brain 126, 470–481. 10.1093/brain/awg044 12538413

[B4] Chen R. Lozano A. M. Ashby P. (1999). Mechanism of the silent period following transcranial magnetic stimulation evidence from epidural recordings. Exp. Brain Res. 128, 539–542. 10.1007/s002210050878 10541749

[B5] Chisari C. (2015). Bottom-up or top-down approach? Understanding the way to reach the milestone of recovery in stroke. Int. J. Neurorehabil. 2:e107 10.4172/2376-0281.1000e107

[B6] Chisari C. Fanciullacci C. Lamola G. Rossi B. Cohen L. G. (2014). NIBS-driven brain plasticity. Arch. Ital. Biol. 152, 247–258. 10.12871/00039829201445 25987184

[B7] Classen J. Schnitzler A. Binkofski F. Werhahn K. J. Kim Y. S. Kessler K. R.. (1997). The motor syndrome associated with exaggerated inhibition within the primary motor cortex of patients with hemiparetic. Brain 120, 605–619. 10.1093/brain/120.4.605 9153123

[B8] Coupar F. Pollock A. Rowe P. Weir C. Langhorne P. (2011). Predictors of upper limb recovery after stroke: a systematic review and meta-analysis. Clin. Rehabil. 26, 291–313. 10.1177/0269215511420305 22023891

[B9] Di Lazzaro V. Profice P. Pilato F. Capone F. Ranieri F. Pasqualetti P.. (2010). Motor cortex plasticity predicts recovery in acute stroke. Cereb. Cortex 20, 1523–1528. 10.1093/cercor/bhp216 19805417

[B10] Di Pino G. Pellegrino G. Assenza G. Capone F. Ferreri F. Formica D.. (2014). Modulation of brain plasticity in stroke: a novel model for neurorehabilitation. Nat. Rev. Neurol. 10, 597–608. 10.1038/nrneurol.2014.162 25201238

[B11] Fugl-Meyer A. R. Jääskö L. Leyman I. Olsson S. Steglind S. (1974). The post-stroke hemiplegic patient. 1. a method for evaluation of physical performance. Scand. J. Rehabil. Med. 7, 13–31. 1135616

[B12] Gerloff C. Bushara K. Sailer A. Wassermann E. M. Chen R. Matsuoka T.. (2006). Multimodal imaging of brain reorganization in motor areas of the contralesional hemisphere of well recovered patients after capsular stroke. Brain 129, 791–808. 10.1093/brain/awh713 16364955

[B13] Gladstone D. J. Danells C. J. Black S. E. (2002). The Fugl-Meyer assessment of motor recovery after stroke: a critical review of its measurement properties. Neurorehabil. Neural Repair 16, 232–240. 10.1177/154596802401105171 12234086

[B14] Harris-Love M. L. Chan E. Dromerick A. W. Cohen L. G. (2016). Neural substrates of motor recovery in severely impaired stroke patients with hand paralysis. Neurorehabil. Neural Repair. 30, 328–338. 10.1177/1545968315594886 26163204 PMC4707136

[B15] Haug B. A. Kukowski B. (1994). Latency and duration of the muscle silent period following transcranial magnetic stimulation in multiple sclerosis, cerebral ischemia, and other upper motoneuron lesions. Neurology 44, 936–936. 10.1212/WNL.44.5.936 8190300

[B16] Hendricks H. T. van Limbeek J. Geurts A. C. Zwarts M. J. (2002). Motor recovery after stroke: a systematic review of the literature. Arch. Phys. Med. Rehabil. 83, 1629–1637. 10.1053/apmr.2002.35473 12422337

[B17] Hummel F. C. Cohen L. G. (2006). Non-invasive brain stimulation: a new strategy to improve neurorehabilitation after stroke? Lancet Neurol. 5, 708–712. 10.1016/S1474-4422(06)70525-7 16857577

[B18] Kukowski B. Haug B. (1991). Quantitative evaluation of the silent period, evoked by transcranial magnetic stimulation during sustained muscle contraction, in normal man and in patients with stroke. Electromyogr. Clin. Neurophysiol. 32, 373–378. 1526218

[B19] Liepert J. (2006). Motor cortex excitability in stroke before and after constraint-induced movement therapy. Cogn. Behav. Neurol. 19, 41–47. 10.1097/00146965-200603000-00005 16633018

[B20] Liepert J. Hamzei F. Weiller C. (2000a). Motor cortex disinhibition of the unaffected hemisphere after acute stroke. Muscle Nerve 23, 1761–1763. 10.1002/1097-4598(200011)23:11<1761::AID-MUS14>3.0.CO;2-M 11054757

[B21] Liepert J. Restemeyer C. Kucinski T. Zittel S. Weiller C. (2005). Motor strokes the lesion location determines motor excitability changes. Stroke 36, 2648–2648. 10.1161/01.STR.0000189629.10603.02 16269647

[B22] Liepert J. Storch P. Fritsch A. Weiller C. (2000b). Motor cortex disinhibition in acute stroke. Clin. Neurophysiol. 111, 671–676. 10.1016/S1388-2457(99)00312-0 10727918

[B23] Mahoney F. I. Barthel D. W. (1965). Functional evaluation: the Barthel index. Md. State Med. J. 14, 61–65. 14258950

[B24] Manganotti P. Patuzzo S. Cortese F. Palermo A. Smania N. Fiaschi A. (2002). Motor disinhibition in affected and unaffected hemisphere in the early period of recovery after stroke. Clin. Neurophysiol. 113, 936–943. 10.1016/S1388-2457(02)00062-7 12048054

[B25] Nowak D. A. Grefkes C. Ameli M. Fink G. R. (2009). Interhemispheric competition after stroke: brain stimulation to enhance recovery of function of the affected hand. Neurorehabil. Neural Repair. 23, 641–656. 10.1177/1545968309336661 19531606

[B26] Nowak D. A. Grefkes C. Dafotakis M. Eickhoff S. Küst J. Karbe H.. (2008). Effects of low-frequency repetitive transcranial magnetic stimulation of the contralesional primary motor cortex on movement kinematics and neural activity in subcortical stroke. Arch. Neurol. 65, 741–747. 10.1001/archneur.65.6.741 18541794

[B27] Pizzi A. Carrai R. Falsini C. Martini M. Verdesca S. Grippo A. (2009). Prognostic value of motor evoked potentials in motor function recovery of upper limb after stroke. J. Rehabil. Med. 41, 654–660. 10.2340/16501977-0389 19565160

[B28] Rossini P. M. Calautti C. Pauri F. Baron J. C. (2003). Post-stroke plastic reorganisation in the adult brain. Lancet Neurol. 2, 493–502. 10.1016/S1474-4422(03)00485-X 12878437

[B29] Schaechter J. D. (2004). Motor rehabilitation and brain plasticity after hemiparetic stroke. Prog. Neurobiol. 73, 61–72. 10.1016/j.pneurobio.2004.04.001 15193779

[B30] Shimizu T. Hosaki A. Hino T. Sato M. Komori T. Hirai S.. (2002). Motor cortical disinhibition in the unaffected hemisphere after unilateral cortical stroke. Brain 125, 1896–1907. 10.1093/brain/awf183 12135979

[B31] Stinear C. M. Byblow W. D. Ward S. H. (2014). An update on predicting motor recovery after stroke. Ann. Phys. Rehabil. Med. 57, 489–498. 10.1016/j.rehab.2014.08.006 25200094

[B32] Swayne O. B. Rothwell J. C. Ward N. S. Greenwood R. J. (2008). Stages of motor output reorganization after hemispheric stroke suggested by longitudinal studies of cortical physiology. Cereb. Cortex 18, 1909–1922. 10.1093/cercor/bhm218 18234688 PMC2474452

[B33] Takechi U. Matsunaga K. Nakanishi R. Yamanaga H. Murayama N. Mafune K.. (2014). Longitudinal changes of motor cortical excitability and transcallosal inhibition after subcortical stroke. Clin. Neurophysiol. 125, 2055–2069. 10.1016/j.clinph.2014.01.034 24636830

[B34] Thickbroom G. W. Cortes M. Rykman A. Volpe B. T. Fregni F. Krebs H. I.. (2015). Stroke subtype and motor impairment influence contralesional excitability. Neurology 85, 517–520. 10.1212/WNL.0000000000001828 26187228 PMC4540249

[B35] Uozumi T. Ito Y. Tsuji S. Murai Y. (1992). Inhibitory period following motor evoked potentials evoked by magnetic cortical stimulation. Electroencephalogr. Clin. Neurophysiol. 85:2739. 10.1016/0168-5597(92)90116-s 1380915

[B36] van Kuijk A. A. Bakker C. D. Hendriks J. C. Geurts A. C. H. Stegeman D. F. Pasman J. W. (2014). Definition dependent properties of the cortical silent period in upper-extremity muscles, a methodological study. J. Neuroeng. Rehabil. 11, 1–9. 10.1186/1743-0003-11-1 24393611 PMC3892048

[B37] van Kuijk A. A. Pasman J. W. Geurts A. C. Hendricks H. T. (2005). How salient is the silent period? The role of the silent period in the prognosis of upper extremity motor recovery after severe stroke. J. Clin. Neurophysiol. 22, 10–24. 10.1097/01.WNP.0000150975.83249.71 15689709

[B38] Ward N. S. Cohen L. G. (2004). Mechanisms underlying recovery of motor function after stroke. Arch. Neurol. 61, 1844–1848. 10.1001/archneur.61.12.1844 15596603 PMC3713312

[B39] Wolf S. L. Catlin P. A. Ellis M. Archer A. L. Morgan B. Piacentino A. (2001). Assessing Wolf motor function test as outcome measure for research in patients after stroke. Stroke 32, 1635–1639. 10.1161/01.STR.32.7.1635 11441212

